# Universal Newborn Screening for Severe Combined Immunodeficiency (SCID)

**DOI:** 10.3389/fped.2019.00373

**Published:** 2019-09-18

**Authors:** Mirjam van der Burg, Nizar Mahlaoui, Hubert Bobby Gaspar, Sung-Yun Pai

**Affiliations:** ^1^Laboratory for Immunology, Department of Pediatrics, Leiden University Medical Center, Leiden, Netherlands; ^2^Centre de Référence Déficits Immunitaires Héréditaires, Hôpital Universitaire Necker-Enfants Malades, Assistance Publique-Hôpitaux de Paris, Paris, France; ^3^Molecular and Cellular Immunology, UCL Great Ormond Street Institute of Child Health, London, United Kingdom; ^4^Division of Hematology-Oncology, Department of Pediatrics, Boston Children's Hospital, Boston, MA, United States; ^5^Department of Pediatric Oncology, Dana-Farber Cancer Institute, Boston, MA, United States; ^6^Department of Pediatrics, Harvard Medical School, Boston, MA, United States

**Keywords:** severe combined immunodeficiency, T lymphocytes, newborn screening, allogeneic hematopoietic stem cell transplantation, public health

## Abstract

Patients with severe combined immunodeficiency (SCID) are born with profound deficiency of functional T-lymphocytes. Early detection and diagnosis would allow for prompt institution of isolation from infection and referral for definitive treatment with allogeneic hematopoietic stem cell transplantation. Universal newborn screening for SCID, using an assay to detect T-cell receptor excision circles (TREC) in dried blood spots (DBS), is now being performed in all states in the United States. In this review, we discuss the development and outcomes of TREC screening, and continued challenges to implementation.

## Screening For SCID With T-cell Receptor Excision Circles (TRECs)

SCID is a severe inherited disorder of the immune system caused by a spectrum of genetic defects leading to cellular and humoral immunodeficiency (1). SCID patients are born asymptomatic, but within the first month of life develop severe (opportunistic) infections, failure to thrive and are vulnerable to secondary infections induced by live vaccines. They generally die before the age of 1 year unless they receive adequate and curative treatment. This includes hematopoietic stem cell transplantation (HSCT) and, in selected genetic forms of SCID, enzyme replacement therapy, or gene therapy.

Fulfillment of principles published by Wilson and Jungner in 1968 (2) remains the foundation to justify addition of new disorders for newborn screening, in order to use public health funds wisely and effectively. In the case of newborn screening for severe combined immunodeficiency (SCID), data were lacking in key areas that hampered the initial fulfillment of those principles, at least in the eyes of public health officials. Single institution experiences argued that early treatment prior to the age of 3.5 months would lead to better survival, but multi-institutional data were lacking (3, 4). Transplantation clearly was the accepted treatment, yet case definitions of SCID and the approach to transplant varied widely. As of the early twenty-first century, the incidence of SCID was estimated at 1 in 100,000 births, yet was potentially an underestimate due to lack of diagnosis.

Furthermore, at that time a suitable test did not yet exist. The success of using dried blood spots (DBS) to measure blood phenylalanine levels and detect phenylketonuria at birth (5) relied on the stability of the analyte, the reproducibility, and low cost of the assay. Therefore, any analyte used for detection of SCID needed to have the same characteristics and be feasible using DBS. Multiple possible analytes were considered, including absolute lymphocyte count or CD3 count by phlebotomy, IL-7 level by immunoassay (high levels correlating with lymphodepletion), and CD3 and CD45 protein by Luminex (6). With data in hand from Jennifer Puck showing that detection of T-cell receptor excision circles (TRECs) was feasible from DBS (7), ultimately the community of disease specialists and newborn screening directors pursued the development of the TREC assay. Concerns and challenges remaining about the TREC assay included feasibility and costs, due to the requirement to extract DNA from all blood specimens.

TRECs are formed as circular excision products during T-cell receptor gene rearrangement in developing T-lymphocytes in the thymus. Therefore, TRECs are a marker for recently formed T-lymphocytes, while conversely, absence or strongly reduced levels of TRECs indicates a low level of newly formed T-lymphocytes. Only some of the children examined for low T cells will in fact have SCID (8). TRECs will not be detected in case of the presence of maternally engrafted T-lymphocytes and also in patients with Omenn Syndrome TRECs will be low/absent because of oligoclonal expansion of the autologous T-lymphocytes, which makes the TREC assay also useful in these subforms of SCID. However, the diagnosis of SCID can only be made after immunophenotyping and can be confirmed by genetic testing.

Low/absent TRECs can also be identified in children with T-lymphocyte impairment syndromes (such as DiGeorge Syndrome, Down's syndrome or Ataxia Telangiectasia), children with T-lymphocyte impairment secondary to other neonatal conditions or patients with idiopathic lymphocytopenia. Furthermore, it should be noted that low/absent TREC levels can also be found in preterm children.

## Implementation of SCID Screening Based on TRECs

Although the TREC assay has been available for over a decade, implementation of this assay in newborn screening (NBS) programs remains a major challenge. In the US, two states received funding from the Center for Disease Control to conduct pilot projects with the goal of determining the feasibility and effectiveness of NBS for SCID using the TREC assay. In Wisconsin, 71,000 infants were screened in 2008, resulting in 11 patients >37 weeks gestation undergoing flow cytometry, and detection of eight infants with T-lymphocytopenia, including an infant with RAC2 mutation who had successful umbilical cord blood HSCT (9). In Massachusetts, 100,597 infants were screened in 2009–2010, resulting in 78 infants (0.08%) undergoing flow cytometry, detection of 29 infants with T-lymphocytopenia, including an infant with SCID due to compound heterozygous mutations in JAK3, who also had successful unrelated donor HSCT (10). Based on the successful detection of T-lymphocytopenia, the Health and Human Services Advisory Committee on Heritable Disorders in Newborns and Children recommended adding SCID to the uniform newborn screening panel, which was announced by HHS Secretary Kathleen Sibelius in May 2010.

As in much of the world, the implementation of TREC NBS proceeded state-by-state, after garnering approval and funding from each state government. By 2013 with screening active in California, New York, Delaware, Michigan, Colorado, Connecticut, Florida, Mississippi, Texas, and the Navajo Nation, approximately 45% of births were screened. Analysis of outcomes of screening in these states and single state experience from California, which screens ~500,000 newborns per year, have yielded several key points. First, as suspected, the incidence of SCID is far higher than previously estimated. In the summary of results from 11 states and the Navajo nation, the incidence of SCID was 1 in 58,000 births based on 52 cases of SCID (42 typical, 9 leaky, 1 Omenn syndrome) in 3,030,083 screened (11). The results of single state experiences are similar; screening in Wisconsin from 2008-2010 yielded 5 SCID patients in 207,696 screened (~1 in 42,000) while screening in California from 2010-2017 yielded 50 cases of SCID in 3,252,156 screened (~1 in 65,000) (8). Second, some of the secondary conditions detected as a result of NBS were unanticipated. By the end of 2018, all states of US implemented NBS for SCID (12).

Outside the US, routine TREC screening is currently performed in Israel, New Zealand, Norway, Taiwan, several provinces in Canada, Switzerland, Germany, Iceland, Sweden, Italy (Tuscany), Spain (Catalunia) and in some regions in Australia. In Israel, 290,864 children were screened between 2015 and 2017 and 13 patients with SCID were identified (~1 in 22,000) (13) whereas only 7 SCID patients were identified between 2010 and 2017 in Taiwan, where 920,398 children were screened (~1 in 131,000). This illustrates a broad range in incidence in the various countries (14).

## Pilot Programs Addressing Additional Aspects of Screening

In several other countries pilot programs have been performed or are under way addressing also other aspects of SCID screening prior to the decision of implementation in national screening programs.

In Sweden, a 2-year pilot study was performed encompassing 58,834 children, which included in addition to TREC screening, KREC screening to identify B-lymphocytopenia concomitantly. KREC refers to the kappa recombination excision circle, which is formed during specific rearrangement in the IGK locus in developing B-lymphocytes (i.e., intron RSS-Kde rearrangement) (15) KRECs could be detected by quantitative PCR similar to TRECs even in a multiplex assay together with a control gene. In the Swedish pilot study, 1 patient with Artemis-SCID was identified, one patient with Ataxia Telangiectasia and one unclassified T-lymphocytopenia/hypogammaglobulinemia. Thirteen children born to mothers treated with immunosuppressive agents during pregnancy showed low KREC levels at birth, which spontaneously normalized (16) TREC/KREC screening was also adopted in the pilot study in Spain (17). The advantage of the combined TREC/KREC assay is that it enabled identification of individuals with different forms of PID, which might be missed by TREC assay, including late-onset ADA, some cases of Nijmegen Breakage syndrome and X-linked or autosomal recessive agammaglobulinemia (18, 19). However, adding KREC to the screening also raises the question, whether screening for congenital agammaglobulinemia fulfills the Wilson and Jungner criteria for screening and whether there is enough evidence that it saves lives and decreases morbidity. This should be addressed in a large retrospective study.

The pilot study in France (DEPISTREC) compared a group of 190,517 babies who underwent TREC screening with a control group of 1,400,000 babies and both clinical and economic aspects were investigated (20, 21). Via TREC screening three patients with SCID were identified vs. 28 in the control group, of whom five children died from SCID before they could receive HSCT, which could have been prevented by TREC screening. The economic evaluation revealed that the main cost determinants are incidence and price per test and that routine SCID screening is feasible and effective (20).

The implementation pilot study in the Netherlands (SONNET) started April 2018 (22) and focuses not only on practical implementation of TREC screening the Dutch neonatal screening program, but also includes analysis of lifetime costs and effects of newborn screening for SCID compared to a situation without screening in the Netherlands in a decision analysis model (23). In addition, ethical, legal and societal impact of the screening on parents and health care providers will be studied. Saudi Arabia has a high incidence of SCID. In a series of 8,718 samples TREC levels were determined and on the 16 samples with low TREC target next generation sequencing of a PID panel (T-NGS PID) was performed on DNA isolated from DBS, which appeared to be new reliable approach in SCID screening (24). T-NGS PID whole exome sequencing (in combination with a SCID filter) is part of follow-up diagnostics in most of the NBS programs (18). The National Institutes of Health is currently evaluating newborn sequencing in genomic medicine and public health (25). However, there are many technical, clinical, ethical, and societal challenges before such technology might be widely adopted in newborn screening programs.

Finally, the United Kingdom National Screening Committee has decided to initiate an evaluation study that is in the late stages of planning and there are hospital pilots in Spain (Andalucia and Madrid), Finland and Brazil. A pilot has been done in South Korea, Belgium (Flandres) is contemplating a pilot and Austria is considering nationwide screening.

## Treatment strategies

The potential impact of NBS on treatment of SCID has been suggested by large registry studies conducted by the Primary Immune Deficiency Treatment Consortium (PIDTC) in North America (funded by National Institutes of Health) (26) and the SCETIDE registry in Europe. The PIDTC showed in a retrospective analysis of 240 patients that infants transplanted at >3.5 months of age who had active infection had only 50% survival, in contrast to infants transplanted at <3.5 months of age who had 94% survival (27), corroborating data from the SCETIDE registry and other studies (28–30). Further, analysis of the 571 recipients of HSCT from donors other than matched sibling donors transplanted at PIDTC centers from 1982 to 2012 confirmed the negative impact of active infection on overall survival (31).

Anticipating that widespread implementation of NBS would result in fewer patients with active infection at HSCT and therefore better survival, the PIDTC compared survival of patients diagnosed by NBS, family history or both, with patients diagnosed by clinical signs (32). Surprisingly, survival of patients diagnosed by NBS or family history was identical to patients diagnosed clinically. This prospective cohort is quite small, with only 98 patients included in the main analyses. Notably the survival of actively infected patients was lower than those without active infection (81 vs. 95%), but high compared to historical larger studies. These results may argue against the fulfillment of the Wilson and Jungner criteria, because early recognition in an asymptomatic state may not improve outcome significantly. However, this study did not reflect on *how* patients survive. Dvorak et al. have addressed this point in a single-center study including 83 SCID receiving HSCT. They found that patients with a positive family history or identified via NBS showed better neurologic event–free survival (NEFS) (33). This indicates that on top of survival, NEFS and other EFS could be important outcomes, best measured in prospective (multi-center) studies.

In addition, there remain other challenges to implementation of NBS. Despite undergoing HSCT at a younger age (78 vs. 239 days), 42% of patients identified by NBS or family history nevertheless experienced infections before HSCT, and 76% of these were identified after confirmation of the SCID diagnosis. This may reflect the wide variation in the timing of return of results of a positive TREC screen, clinical immunology evaluation, diagnosis of SCID, and implementation of protective measures against infection. CMV infection was prevalent, affecting 4 of 11 patients who died, raising the question of how to prevent CMV acquisition e.g., via breastfeeding (34). Analysis of a larger cohort and of similar studies in other countries will be key to fulfilling the promise of NBS.

The optimal HSCT approach for these patients remains unclear, and likely should be tailored to the genotype of SCID, which is associated with survival, the likelihood of T-lymphocyte reconstitution, as well as humoral immune function after HSCT (31). A PIDTC trial randomizing NBS SCID patients of certain genotypes to 2 different busulfan-based regimens is one way that optimal approaches can be identified (NCT03619551) (see review on “HSCT in Severe Combined Immunodeficiency”).

## Summary and Future Perspectives

NBS for SCID based on measurement of TRECs allows early identification, protection from infection, and early treatment, all anticipated to increase overall survival. Implementation will proceed as soon as the required pilot studies has demonstrated the added value of screening for SCID patients and cost effectiveness. Emerging experience in the United States suggests that avoiding acquisition of infection after SCID is diagnosed remains crucial to impact on survival. Close partnership of NBS programs, immunologists, and transplant specialists in each region and sharing of experiences internationally would be ideal to promote standardization of care and identification of best practices.

- TREC screening allow identification of SCID patients in the asymptomatic phase of the disease and early treatment.
- TREC screening is implemented in US and several other countries and is expected to be implemented in many more countries.
- Preventing identified newborns with SCID from acquiring infection remains important and challenging.
- Development of best practices for HCT approach to SCID patients identified by NBS is ongoing.

## Author Contributions

MB, NM, HG, and S-YP conceived the concept of the chapter, wrote the manuscript, and revised the final manuscript.

### Conflict of Interest Statement

The authors declare that the research was conducted in the absence of any commercial or financial relationships that could be construed as a potential conflict of interest.
